# Supplementary material for: Cntnap2 loss drives striatal neuron hyperexcitability and behavioral inflexibility
Source: eLife. 2025 Jul 21;13:RP100162. doi: 10.7554/eLife.100162 (PMC12279377; doi:10.7554/eLife.100162)
Supplement: Figure 2—figure supplement 2—source data 1. [file elife-100162-fig2-figsupp2-data1.zip › Figure_2-figure_supplement_2-source_data_1.pdf]

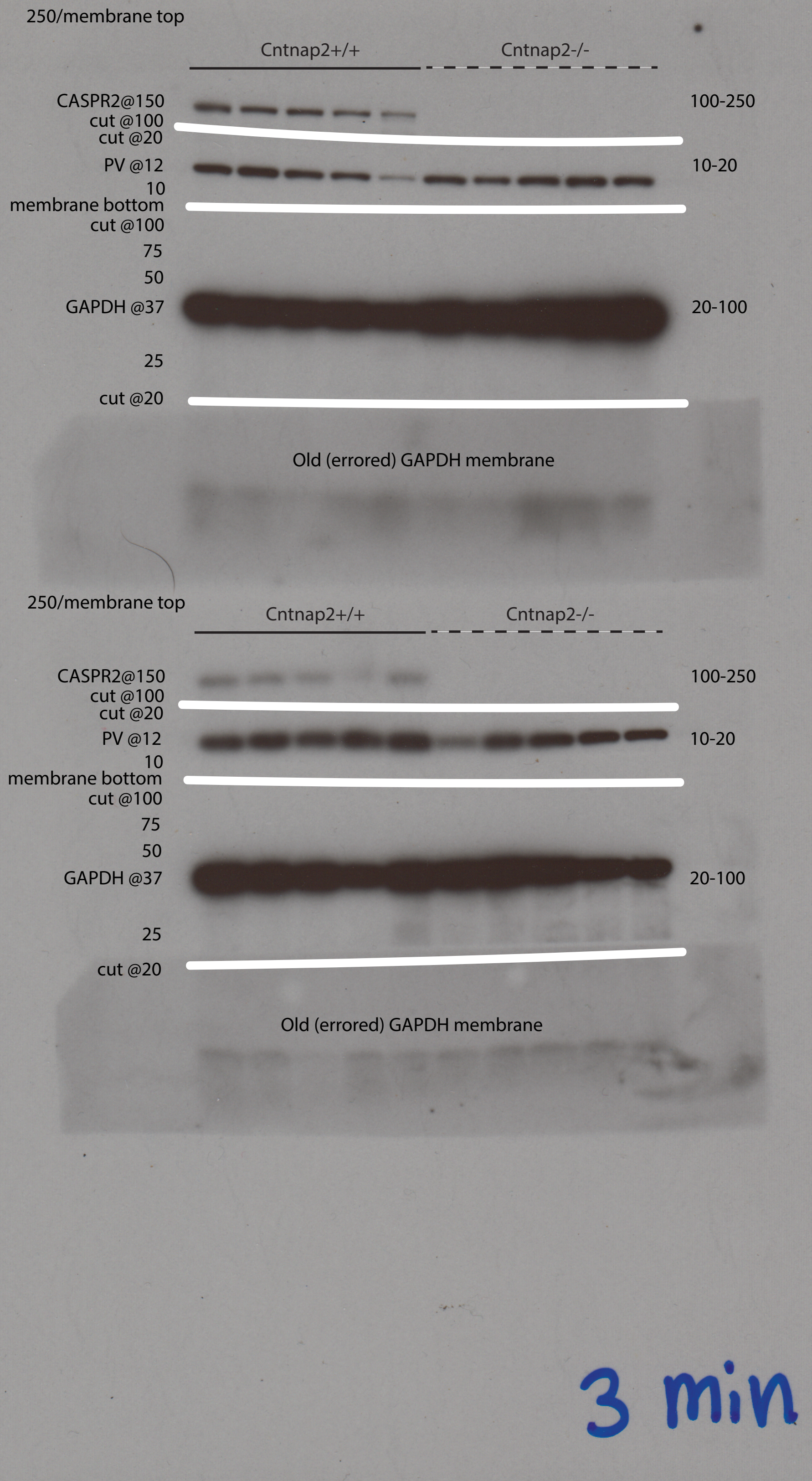
250/membrane top
Cntnap2+/+
Cntnap2-/-
CASPR2@150
cut @100
cut @20
PV @12
10
membrane bottom
cut @100
75
50
GAPDH @37
25
cut @20
100-250
10-20
20-100
Old (errored) GAPDH membrane
250/membrane top
Cntnap2+/+
Cntnap2-/-
CASPR2@150
cut @100
cut @20
PV @12
10
membrane bottom
cut @100
75
50
GAPDH @37
25
cut @20
100-250
10-20
20-100
Old (errored) GAPDH membrane
3 min
