# Supplementary figures and images for: Cntnap2 loss drives striatal neuron hyperexcitability and behavioral inflexibility
Source: eLife. 2025 Jul 21;13:RP100162. doi: 10.7554/eLife.100162 (PMC12279377; doi:10.7554/eLife.100162)

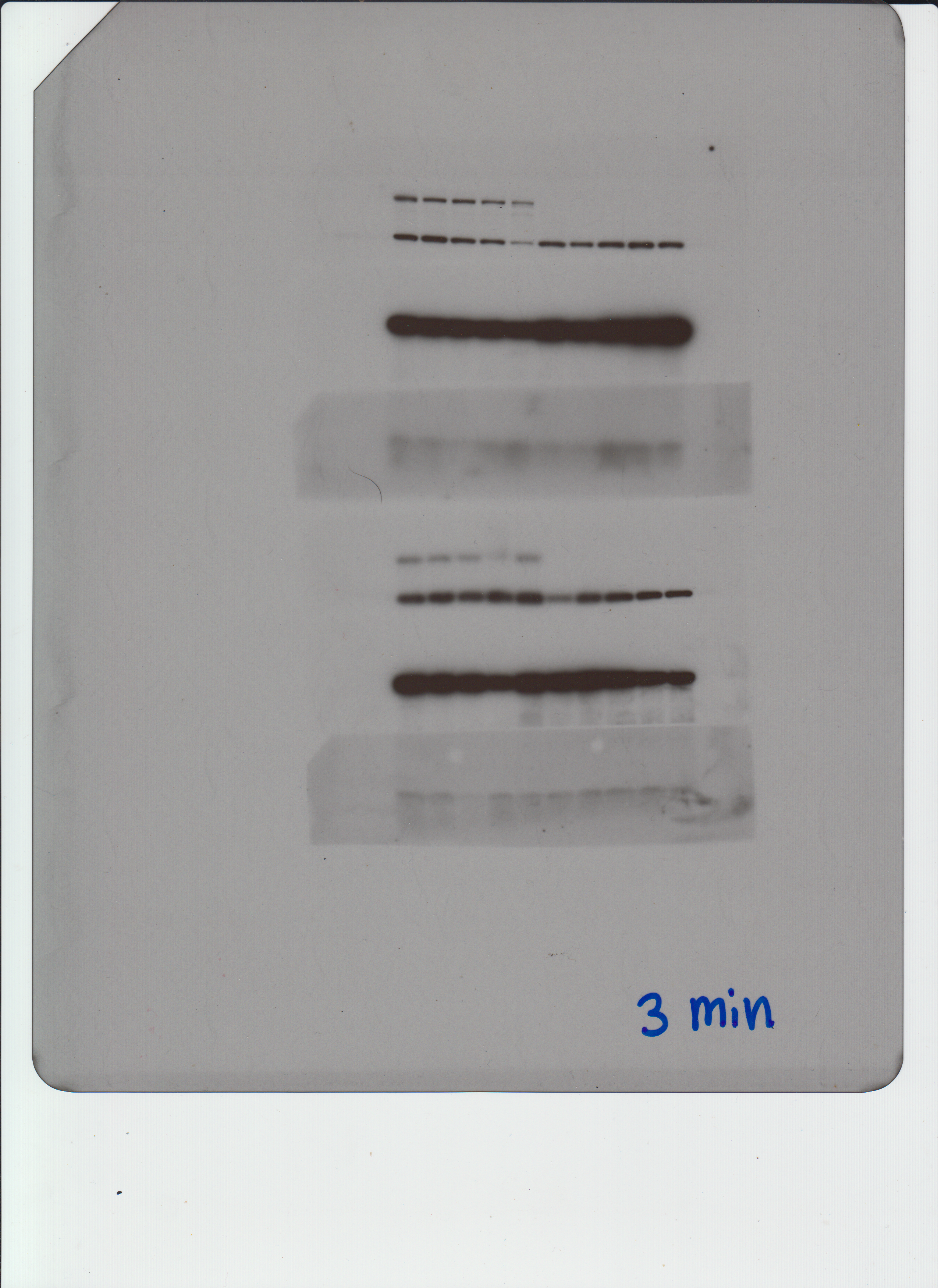

Supplement: Figure 2—figure supplement 2—source data 2. [file elife-100162-fig2-figsupp2-data2.zip › Figure_2-figure_supplement_2-source_data_2.tiff]
