# Supplementary material for: Cntnap2 loss drives striatal neuron hyperexcitability and behavioral inflexibility
Source: eLife. 2025 Jul 21;13:RP100162. doi: 10.7554/eLife.100162 (PMC12279377; doi:10.7554/eLife.100162)
Supplement: Supplementary file 1. — Table reporting the behavior test results for each assay, separated by sex and genotype. [file elife-100162-supp1.docx]

**Supplementary File 1. Summary of behavior data by sex and genotype**

| **Group** | **N**  **(mice)** | **Mean** | **Std. deviation** | **Std. error of the mean** |
| --- | --- | --- | --- | --- |
| ***Open field*** |  |  |  |  |
| *Cntnap2^+/+^* male distance traveled | 22 | 127.7 m | 54.34 | 11.58 |
| *Cntnap2^-/-^* male distance traveled | 16 | 156 m | 28.36 | 7.091 |
| *Cntnap2^+/+^* female distance traveled | 19 | 139.2 m | 48.71 | 11.17 |
| *Cntnap2^-/-^* female distance traveled | 18 | 131.7 m | 57.52 | 13.56 |
| *Cntnap2^+/+^* male avg. speed | 22 | 0.03536 m/s | 0.01500 | 0.003198 |
| *Cntnap2^-/-^* male avg. speed | 16 | 0.04331 m/s | 0.007964 | 0.001991 |
| *Cntnap2^+/+^* female avg. speed | 19 | 0.03868 m/s | 0.01341 | 0.003076 |
| *Cntnap2^-/-^* female avg. speed | 18 | 0.03644 m/s | 0.01590 | 0.003749 |
| *Cntnap2^+/+^* male rears | 22 | 557.6 rears | 201.8 | 43.03 |
| *Cntnap2^-/-^* male rears | 16 | 462.8 rears | 173.0 | 43.25 |
| *Cntnap2^+/+^* female rears | 19 | 361.2 rears | 188.3 | 43.19 |
| *Cntnap2^-/-^* female rears | 18 | 355.1 rears | 158.8 | 37.42 |
| *Cntnap2^+/+^* male center entries | 22 | 254.3 entries | 123.3 | 26.29 |
| *Cntnap2^-/-^* male center entries | 16 | 350.8 entries | 102.4 | 25.59 |
| *Cntnap2^+/+^* female center entries | 19 | 204.8 entries | 71.51 | 16.41 |
| *Cntnap2^-/-^* female center entries | 18 | 247.6 entries | 88.30 | 20.81 |
| *Cntnap2^+/+^* male number of grooming bouts (20 min.) | 22 | 14.91 bouts | 8.602 | 1.834 |
| *Cntnap2^-/-^* male number of grooming bouts (20 min.) | 16 | 19.56 bouts | 11.52 | 2.879 |
| *Cntnap2^+/+^* female number of grooming bouts (20 min.) | 19 | 13.37 bouts | 6.353 | 1.457 |
| *Cntnap2^-/-^* female number of grooming bouts (20 min.) | 18 | 28.44 bouts | 18.99 | 4.475 |
| ***Marble burying*** |  |  |  |  |
| *Cntnap2^+/+^* male marbles buried | 16 | 8.125 marbles | 6.206 | 1.552 |
| *Cntnap2^-/-^* male marbles buried | 17 | 10.82 marbles | 5.187 | 1.258 |
| *Cntnap2^+/+^* female marbles buried | 17 | 8.235 marbles | 5.641 | 1.368 |
| *Cntnap2^-/-^* female marbles buried | 16 | 11.25 marbles | 5.310 | 1.328 |
| ***Holeboard*** |  |  |  |  |
| *Cntnap2^+/+^* male 10 min. nose pokes | 11 | 156.5 pokes | 21.18 | 6.385 |
| *Cntnap2^-/-^* male 10 min. nose pokes | 11 | 192.3 pokes | 38.79 | 11.70 |
| *Cntnap2^+/+^* female 10 min. nose pokes | 14 | 175.9 pokes | 37.51 | 10.03 |
| *Cntnap2^-/-^* female 10 min. nose pokes | 11 | 180.5 pokes | 47.50 | 14.32 |
| *Cntnap2^+/+^* male first 5 min. nose pokes | 11 | 105.4 pokes | 16.26 | 4.901 |
| *Cntnap2^-/-^* male first 5 min. nose pokes | 11 | 114.5 pokes | 30.25 | 9.121 |
| *Cntnap2^+/+^* female first 5 min. nose pokes | 14 | 110.5 pokes | 22.93 | 6.128 |
| *Cntnap2^-/-^* female first 5 min. nose pokes | 11 | 112.3 pokes | 30.09 | 9.073 |
| *Cntnap2^+/+^* male last 5 min. nose pokes | 11 | 51.18 pokes | 10.09 | 3.042 |
| *Cntnap2^-/-^* male last 5 min. nose pokes | 11 | 77.73 pokes | 14.80 | 4.462 |
| *Cntnap2^+/+^* female last 5 min. nose pokes | 14 | 65.36 pokes | 20.82 | 5.565 |
| *Cntnap2^-/-^* female last 5 min. nose pokes | 11 | 68.27 pokes | 20.15 | 6.077 |
| ***Rotarod*** |  |  |  |  |
| *Cntnap2^+/+^* male learning rate | 15 | 1.839 RPM/day | 1.416 | 0.3655 |
| *Cntnap2^-/-^* male learning rate | 14 | 2.856 RPM/day | 1.166 | 0.3115 |
| *Cntnap2^+/+^* female learning rate | 15 | 2.162 RPM/day | 1.083 | 0.2798 |
| *Cntnap2^-/-^* female learning rate | 15 | 3.607 RPM/day | 1.103 | 0.2849 |
| ***Four choice reversal learning*** |  |  |  |  |
| *Cntnap2^+/+^* male acquisition trials | 5 | 20.80 trials | 6.834 | 3.056 |
| *Cntnap2^-/-^* male acquisition trials | 5 | 20 trials | 7.106 | 3.178 |
| *Cntnap2^+/+^* female acquisition trials | 5 | 17.20 trials | 4.550 | 2.035 |
| *Cntnap2^-/-^* female acquisition trials | 5 | 19.60 trials | 2.302 | 1.030 |
| *Cntnap2^+/+^* male recall trials | 5 | 11 trials | 1.871 | 0.8367 |
| *Cntnap2^-/-^* male recall trials | 5 | 11 trials | 4.243 | 1.897 |
| *Cntnap2^+/+^* female recall trials | 5 | 10.60 trials | 1.140 | 0.5099 |
| *Cntnap2^-/-^* female recall trials | 5 | 10.20 trials | 1.095 | 0.4899 |
| *Cntnap2^+/+^* male reversal trials | 5 | 18.40 trials | 1.817 | 0.8124 |
| *Cntnap2^-/-^* male reversal trials | 5 | 30.40 trials | 4.037 | 1.806 |
| *Cntnap2^+/+^* female reversal trials | 5 | 26.40 trials | 9.737 | 4.354 |
| *Cntnap2^-/-^* female reversal trials | 5 | 32.60 trials | 4.827 | 2.159 |
| *Cntnap2^+/+^* male reversal errors | 5 | 7.400 errors | 0.8944 | 0.4000 |
| *Cntnap2^-/-^* male reversal errors | 5 | 17.60 errors | 2.608 | 1.166 |
| *Cntnap2^+/+^* female reversal errors | 5 | 12.20 errors | 6.380 | 2.853 |
| *Cntnap2^-/-^* female reversal errors | 5 | 15.20 errors | 1.789 | 0.8000 |
| ***DeepLabCut Keypoint-MoSeq Open Field*** |  |  |  |  |
| *Cntnap2^+/+^* male distance traveled | 7 | 272 m | 6112 | 2310 |
| *Cntnap2^-/-^* male distance traveled | 5 | 232 m | 4876 | 2181 |
| *Cntnap2^+/+^* female distance traveled | 6 | 202 m | 1604 | 654.9 |
| *Cntnap2^-/-^* female distance traveled | 6 | 227 m | 4651 | 1899 |
| *Cntnap2^+/+^* male number of grooming bouts (60 min.) | 7 | 324.7 bouts | 166.1 | 62.77 |
| *Cntnap2^-/-^* male number of grooming bouts (60 min.) | 5 | 411.6 bouts | 131.6 | 58.84 |
| *Cntnap2^+/+^* female number of grooming bouts (60 min.) | 6 | 361.0 bouts | 92.18 | 37.63 |
| *Cntnap2^-/-^* female number of grooming bouts (60 min.) | 6 | 534.0 bouts | 206.9 | 84.47 |
